# Biodegradable Alginate Films with ZnO Nanoparticles and Citronella Essential Oil—A Novel Antimicrobial Structure

**DOI:** 10.3390/pharmaceutics13071020

**Published:** 2021-07-03

**Authors:** Ludmila Motelica, Denisa Ficai, Ovidiu Oprea, Anton Ficai, Roxana-Doina Trusca, Ecaterina Andronescu, Alina Maria Holban

**Affiliations:** 1 Faculty of Applied Chemistry and Materials Science, University Politehnica of Bucharest, Spl. Independentei 313, 060042 Bucharest, Romania; motelica_ludmila@yahoo.com (L.M.); denisa.ficai@upb.ro (D.F.); anton.ficai@upb.ro (A.F.); truscaroxana@yahoo.com (R.-D.T.); ecaterina.andronescu@upb.ro (E.A.); alina.m.holban@bio.unibuc.ro (A.M.H.); 2 Academy of Romanian Scientists, Ilfov st. 3, 050045 Bucharest, Romania; 3 Microbiology & Immunology Department, Faculty of Biology, University of Bucharest, 077206 Bucharest, Romania

**Keywords:** biodegradable, alginate film, antibacterial packaging, citronella essential oil, zinc oxide nanoparticles, edible packaging, cheese

## Abstract

The petroleum-based materials could be replaced, at least partially, by biodegradable packaging. Adding antimicrobial activity to the new packaging materials can also help improve the shelf life of food and diminish the spoilage. The objective of this research was to obtain a novel antibacterial packaging, based on alginate as biodegradable polymer. The antibacterial activity was induced to the alginate films by adding various amounts of ZnO nanoparticles loaded with citronella (lemongrass) essential oil (CEO). The obtained films were characterized, and antibacterial activity was tested against two Gram-negative (*Escherichia coli* and *Salmonella* Typhi) and two Gram-positive (*Bacillus cereus* and *Staphylococcus aureus*) bacterial strains. The results suggest the existence of synergy between antibacterial activities of ZnO and CEO against all tested bacterial strains. The obtained films have a good antibacterial coverage, being efficient against several pathogens, the best results being obtained against *Bacillus cereus*. In addition, the films presented better UV light barrier properties and lower water vapor permeability (WVP) when compared with a simple alginate film. The preliminary tests indicate that the alginate films with ZnO nanoparticles and CEO can be used to successfully preserve the cheese. Therefore, our research evidences the feasibility of using alginate/ZnO/CEO films as antibacterial packaging for cheese in order to extend its shelf life.

## 1. Introduction

Cheese, one of the most popular foods, is made of casein, fat and water, but unfortunately, when it is not salted, it has a limited shelf life. Extensive and uncontrolled microbial development on its surface is leading to the short shelf life of this kind of cheese. The need of bacterial cultures to produce the cheese varieties creates an infection hazard with cheese-borne species like *Escherichia coli*, *Salmonella enterica* or *Staphylococcus aureus* [1]. At present, the majority of food packaging used in industry is based on petrochemical polymers or cellulose due to low-cost and good mechanical and barrier performances [2]. Environmental concerns are building up pressure in favor of biodegradable packaging, from renewable sources like cellulose-based materials [3]. The majority of plastics used as packaging are not biodegradable and represent an environmental hazard worldwide increasing short- and long-term pollution [4]. Nevertheless, neither cellulose nor plastic packaging materials present antimicrobial activity. The environmental factors and the need to decrease food losses are constantly pressuring the food packaging industry to develop new types of antimicrobial and biodegradable materials. Such innovative materials can actively control the microorganisms’ proliferation, reducing the food loss by spoilage, prolonging the shelf life and in the end ensuring a better food quality and safety [5,6,7,8].

Sodium alginate (A) is the salt of alginic acid, which belongs to polysaccharides and as such is a biocompatible and biodegradable polymer. Most often, the alginate is obtained from marine algae. The polymeric structure is composed from α-l-guluronate (G) and β-d-mannuronate (M) units linked by β-1-4 glycosidic bond, which can form MM, GG or MG blocks [9]. Alginate is water-soluble, can be easily handled and functionalized [10], and is a natural choice for active packaging due to its lack of toxicity [11,12], US-FDA considering it as GRAS (Generally Recognized As Safe) [13]. Plasticizers are usually added to improve some of the mechanical properties of alginate films, glycerol being most often used due to its good compatibility [14]. Mechanical properties can be enhanced furthermore by reinforcing the biopolymer with nanoparticles (NPs), their inclusion improving also the barrier properties. Some nanoparticles, e.g., ZnO, will act like crosslinking agents when they are embedded into the alginate matrix [15,16].

Some of the properties of ZnO lead to applications that make it one of the most studied nanomaterials. ZnO has a high absorbance under 380 nm and therefore is often used as sunscreen, and it is used as a biocide in the medicine or food industry due to its potent antimicrobial activity [17,18,19,20,21]. Like many other nanoscale materials, ZnO nanoparticles present a large surface area, good loading capacity and potent antimicrobial activity [5]. While many papers explored the antibacterial activity of ZnO, the antimycotic activity is seldom investigated [22,23,24,25,26]. The antimicrobial mechanism supposedly involves two separate pathways, in the presence of light and in the dark. The strong antimicrobial activity exhibited in the presence of light is due to reactive oxygen species (ROS) production, ZnO presenting a good photocatalytic activity. The ROS are responsible for the antibacterial activity, as they will damage the bacterial membrane by oxidative stress. In the absence of light the antibacterial activity is smaller, but still noticeable, the most probable mechanisms involving nanoparticle internalization and some mechanical damage, like puncture or rupture of the bacterial cellular wall [27,28,29,30]. Very important, US-FDA consider ZnO as GRAS and acceptable as an active component in food packaging due to its good antimicrobial properties [31,32,33].

Essential oils and other plant extracts present a huge potential as antibacterial and antioxidant agents and therefore are the subject of many research studies [34,35,36,37]. The major constituents of citronella essential oil (CEO), among other substances, are citral, citronellal and citronellol [38,39]. CEO is considered a non-toxic biopesticide in the US [40] which also has a very strong antifungal and antibacterial activity [41,42,43]. The bare alginate films exhibit a poor performance regarding water vapor permeability (WVP). In order to decrease the hydrophilicity of the alginate films, hydrophobic plant extracts or essential oils can be added into the composition [44,45,46].

The introduction of ZnO nanoparticles into the food packaging will enhance the antibacterial activity [14] and will improve the UV-barrier of the polymeric film. Both qualities will extend the shelf life of the packed food [47], but there is an associated concern about the risks posed by the potential migration of NPs into the food, and further into the human body. The antibacterial activity of the NPs comes from the interaction with microbial cells, but at the same time the ZnO NPs can interact with human cells. In order to enhance the antibacterial activity of the packaging films, the ZnO can be loaded with other potent agents [48,49,50]. The use of a natural antimicrobial agent, like an essential oil, along with ZnO permits the use of low concentration for nanoparticles due to the synergic action. When ZnO nanoparticles are used as antibacterial agent the concentration is small enough to be considered harmless, but safety issues are increased if ZnO is used for a long time, due to accumulation and higher concentrations being obtained [51]. Special concerns are related to the nano form when ingested [52]. Studies presenting ZnO nanoparticles cytotoxicity if ingested or applied onto human skin are reported [53,54,55] as a consequence of their widespread uses. While the information obtained on human exposure in realistic uses remains scarce [56], the results reported for the cytotoxicity of ZnO NPs at concentrations that are much higher than would be expected for normal exposure (topical, ingestion or inhalation) [57] remain controversial. Furthermore, negative results were obtained for all in vivo and the majority of in vitro genotoxicity tests for ZnO NPs [58,59,60]. While small ZnO quantities upon ingestion can be assimilated as a beneficial Zn^2+^ source, more studies are needed in order to assess its safety for long-term exposure [61]. At the used CEO concentrations the studies report no toxicity, but some concerns exist about the possible impact on beneficial gut bacteria [62,63].

Our objective in this research was to obtain an antibacterial, biodegradable packaging material, capable of extending food shelf life. As a model, we chose a traditional sort of cheese that usually can be kept only for 4 days in the fridge (4–8 °C) after packaging is opened. The literature presents many reports of alginate packaging for various cheese assortments, especially in recent years [13,64,65,66,67]. Some contain essential oils or other plant extracts as antimicrobial agents [68,69]. While there are reports for chitosan or cellulosic edible films with ZnO nanoparticles for cheese packaging [70,71], in the alginate case none of them contain ZnO. There are few reports of alginate–ZnO packaging films for fruits [14,72]. Therefore, alginate was chosen as the film forming biopolymer, in which ZnO nanoparticles were added as filler and antibacterial agent. In order to enhance the antibacterial activity of the nanocomposite films, the ZnO nanoparticles were loaded with citronella essential oil (CEO). To the best of our knowledge, this is the first time when an alginate-based packaging with ZnO NPs loaded with citronella essential oil is obtained. The biological tests against two Gram-positive and two Gram-negative bacterial strains indicate that a synergic effect was obtained between ZnO and CEO, the nanocomposite films presenting a good antibacterial activity against all four strains.

## 2. Materials and Methods

### 2.1. Materials

Zinc acetate dihydrate with 99.9% purity and absolute ethanol were obtained from Merck. Sodium alginate (CAS 9005-38-3) was purchased from Fisher Scientific U.K. Ltd. (Redox Lab Supplies, Bucharest, Romania). Phosphate-buffered saline (PBS), Glycerol, Nutrient Broth, and agar were obtained from Sigma Aldrich (Redox Lab Supplies, Bucharest, Romania). Citronella essential oil (CEO) was purchased from Carl Roth (Amex-Lab, Bucharest, Romania). All the chemicals were used without any further purification.

The soft telemea cheese (S.C. Fabrica de lapte Brasov S.A., Halchiu, BV, Romania) was obtained from a local supermarket in Bucharest, Romania.

### 2.2. Synthesis of ZnO Nanoparticles

ZnO synthesis was performed as described previously in [73]. Briefly, 2.1950 g Zn(CH_3_COO)_2_∙2H_2_O was dissolved in 50 mL absolute ethanol and heated at boiling point under magnetic stirring for 10 h. The obtained precipitate was washed twice with water and finally with ethanol. The separation was done by centrifugation and the resulting powder was dried at 105 °C.

### 2.3. Synthesis of Alginate/ZnO/CEO Films

A certain amount of ZnO NPs were suspended in 10 mL water and mixed with 1 mL CEO. The obtained suspension was sonicated for 30 min before being used to prepare AZ1-AZ4 films (Table 1).

Alginate films were obtained by solvent casting method. In total, 3 g alginate was added in 100 mL water and left to dissolve under stirring for 24 h. Then, 2 mL of glycerol was added to the obtained alginate solution. The previously prepared ZnO&CEO suspension was added to the alginate solution, under vigorous stirring.

Each solution was put in a Petri dish and was left to dry in an oven for 24 h at 40 °C. A control film without ZnO NPs and CEO was prepared in the same way. After film drying, 200 mL CaCl_2_ solution (0.2 M) was added to each Petri-dish and the films were left submerged for 5 min. The films were removed from the Petri-dish and were stored in zip lock plastic bags at 20 °C and 60% relative humidity (RH).

### 2.4. Characterization of Alginate Composite Films

#### 2.4.1. Microstructural Analysis

In order to investigate the films surface morphology and microstructure scanning electron micrographs were obtained using a QUANTA INSPECT F50 scanning electron microscope (FEI Company, Eindhoven, The Netherlands) equipped with field emission gun—FEG with 1.2 nm resolution and an energy dispersive X-ray spectrometer (EDS) with an MnK resolution of 133 eV. Kα lines, specific to each element, were used to map the distribution of C, O and Zn across the sample’s surface.

#### 2.4.2. Fourier Transform Infrared Spectroscopy

By using Fourier transform infrared spectroscopy (FTIR) in the wavenumber range 4000–400 cm^−1^ we investigated the presence of certain functional groups and the interactions between some components of the nanocomposite films. A Nicolet iS50 FTIR spectrometer (Thermo Fisher Scientific Inc., Waltham, MA, USA), equipped with a DTGS detector, was used to record the spectra. We used a resolution of 4 cm^−1^, and each spectra was obtained by averaging 32 scans.

FTIR 2D maps were recorded with a Nicolet iS50R FTIR microscope (Thermo Fisher Scientific Inc., Waltham, MA, USA), with DTGS detector, in the wavenumber range 4000–600 cm^−1^. The 2D FTIR maps were used to obtain information about the spatial distribution of the components

#### 2.4.3. Photoluminescence Spectroscopy

A Perkin Elmer (Waltham, MA, USA) LS55 spectrometer was used to measure the photoluminescence spectrum (PL). The configuration is using a Xe lamp as a UV light source at ambient temperature, the fluorescence being measured in the range 350–800 nm. The spectra were recorded with a scan speed of 200 nm min^−1^, excitation and emission slits of 10 nm, and a 1% attenuator (only 1% of the actual fluorescence is recorded, as the full emission is exceeding the spectrometer scale). An excitation wavelength of 320 nm was used.

#### 2.4.4. UV-Vis Spectroscopy

A JASCO V560 spectrophotometer (JASCO Inc., Easton, PA, USA) was used to measure the UV–Vis spectra. The device was equipped with a 60 mm integrating sphere (ISV-469) and a film holder for the samples. The spectra were recorded with a speed of 200 nm min^−1^, in the domain 200–900 nm.

The opacity values were calculated as A_600_/*x* = −logT_600_/*x*, where A_600_ is the absorbance at 600 nm, T_600_ is the fractional transmittance at 600 nm and *x* is the film thickness in mm. A higher opacity value indicates that the film is less transparent [74].

#### 2.4.5. Thermal Analysis

Thermal analysis, TG-DSC, was performed with a STA 449C Jupiter apparatus, from Netzsch (Selb, Germany). Each sample weighed approximatively 10 mg. The samples were placed in an open alumina crucible and heated up to 900 °C with 10 K∙min^−1^ rate, under flow of 50 mL∙min^−1^ dried air. As reference, we used an empty alumina crucible. The evolved gases were analyzed with a FTIR Tensor 27 from Bruker (Bruker Co., Ettlingen, Germany), equipped with a thermostated gas cell.

#### 2.4.6. Water Vapor Permeability (WVP)

For the determination of water vapor permeability (WVP) we used permeation cups with a diameter of 30 mm, sealed with a sample film, as described in [75]. In each cup we placed 1 g of dried CaCl_2_. The permeation cups were placed in a container at a temperature of 25 °C and 100% relative humidity. Their weight was measured at fix intervals (8 h) for four days.

#### 2.4.7. Swelling Capacity

The swelling capacity was determined as described in [76]. Shortly, square samples of ~3 × 3 cm were cut from the fresh films and were dried in a desiccator for 48 h. Once dried, the samples were weighed (±0.2 mg) (W_0_), then placed in 200 mL water or phosphate buffer saline (PBS) to allow swelling. The samples were weighted at each 30 min for three hours, and at 24 h intervals for next two days as the maximum swelling capacities were attained. The Equation (1) formula for degree of swelling (D) was used to calculate the swelling ratio:

D = (W_t_ − W_0_)/W_0_ (1)

### 2.5. Antibacterial Assay

The antibacterial activity was evaluated against model Gram-positive (*Bacillus cereus* ATCC 13,061 and *Staphylococcus aureus* ATCC 25923) and Gram-negative (*Escherichia coli* ATCC 25,922 and *Salmonella enterica* Typhi ATCC 14023) bacteria, which are relevant in food bacterial contamination. The strains were maintained as glycerol stocks at −80 °C. All experiments were designed and performed in triplicate.

#### 2.5.1. Antibacterial Qualitative Assessment—Growth Inhibition

To qualitatively screen the antibacterial effect of the obtained materials, we utilized an adapted diffusion assay, respecting the general rules exposed in the CLSI 2020 and in our recent study [77]. The obtained 0.5 McFarland bacterial suspensions (1.5 × 10^8^ CFU/mL), previously prepared in sterile saline (0.9% NaCl solution) were utilized as a standardized inoculum to swab inoculate Petri dishes containing nutritive agar. The obtained materials were cut as discs of 6 mm diameter and sterilized by UV exposure for 30 min before use. Sample discs were aseptically placed on the inoculated Petri dishes and these were incubated for 20 h at 37 °C. After incubation, the diameter of growth inhibition developed around each material specimen was measured and expressed in mm.

#### 2.5.2. Evaluation of the Planktonic Development of Microorganisms

Planktonic growth in the presence of the obtained materials was analyzed in nutritive broth. Specimens of 6 mm in diameter were placed in sterile 24-well plates. Then, 1 mL of nutritive broth and 10 μL of the previously obtained 0.5 McFarland bacterial suspensions in PBS were added. Specimens were allowed to incubate for 24 h at 37 °C. To spectrophotometrically evaluate the growth of planktonic (free-floating) cultures, 150 μL of the obtained bacterial culture were transferred to 96-well plates and the absorbance at 600 nm was evaluated.

#### 2.5.3. Monospecific Biofilm Development

The antibiofilm efficiency was established by transferring specimens (sterile, 6 mm in diameter) in sterile 24-well plates containing 1 mL nutritive broth, followed by the inoculation of 10 μL of bacterial suspension of 0.5 McFarland standard density. The as prepared plates were incubated for 24 h at 37 °C. Afterwards the samples were gently washed with 1 mL of sterile saline. In the end, the samples were transferred in 1.5 mL centrifuge tubes, in 1000 μL sterile saline solution. The as obtained samples were then vortexed for 30 s to ensure the detachment of biofilm cells in suspension. After this, serial 10 fold dilutions were obtained and then inoculated on nutrient agar in order to evaluate the viable colony formation, expressed as CFU (colony forming units)/mL.

### 2.6. Preliminary Tests of AZ1-AZ4 Films as Packaging Material for Cheese

Cheese samples (~cubic shape with size of 2 cm) were packed in alginate and AZ1-AZ4 films and placed in a refrigerator (4 °C ± 1 °C and 75% relative humidity) for 14 days. Samples were taken out at 1, 4, 7, 10 and 14 days for the weight loss test. Weight loss was monitored by measuring the weight change of each sample and was calculated as percentage lost from the initial mass.

### 2.7. Statistical Analysis

The obtained results were statistically analyzed using the analysis of variance (ANOVA) performed with Microsoft Excel 2016 (Microsoft Corp., Redmond, WA, USA), having installed XLSTAT 2020.5.1 add-on. The Shapiro–Wilk test was used to check the normal distribution of the data; by Levene’s test we assessed the homoscedasticity of the residuals; the results were compared by Tukey’s (HSD) test so that the pairs of films that differed in terms of statistical significance were revealed (where *p* < 0.05).

## 3. Results and Discussion

### 3.1. Alginate Films Characterization

**Figure 1:**
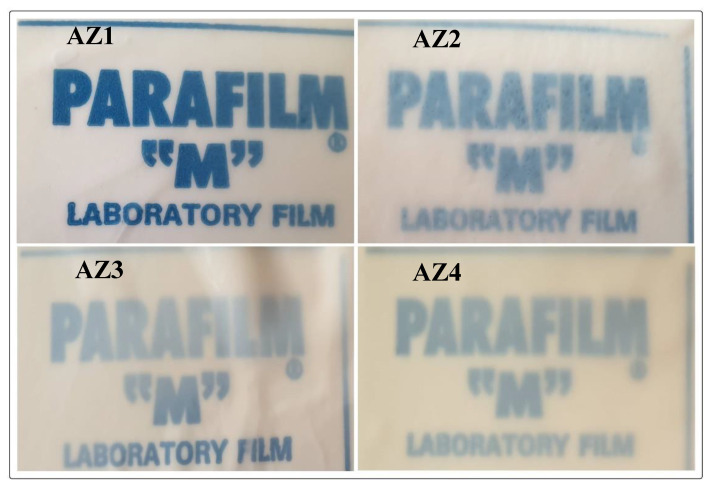
The transparency for AZ1–AZ4 films.

The films were transparent to light yellow from the interaction between alginate, ZnO nanoparticles and CEO. The transparency of the films decreases with the increase of ZnO quantity. In the same series the color intensifies to light yellow (Figure 1).

**Figure 2:**
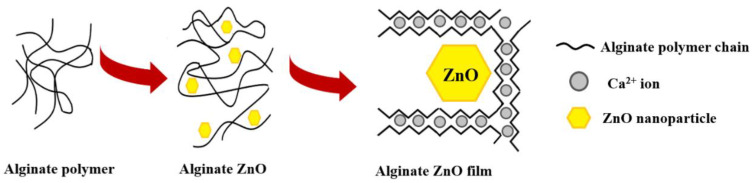
The schematic structure of alginate-ZnO films. The presence of ZnO nanoparticles arranges the alginate polymer chains around them. The addition of calcium ions further strengthens the films according to egg-box model.

The ZnO nanoparticles act as a reinforcing agent for the alginate films and at the same time will arrange the alginate polymeric chains in a more orderly manner. The submerging of alginate/ZnO/CEO films in CaCl_2_ solution will allow calcium ions to penetrate the films and according to the egg-box model [78] to self-assemble the polymeric chains (Figure 2).

Similar samples from all films were analyzed by means of UV-Vis, PL, FTIR, TG/DSC, SEM and antibacterial activity was determined.

### 3.2. UV–Vis and PL Spectrometry

#### 3.2.1. UV–Vis Spectrometry

**Figure 3:**
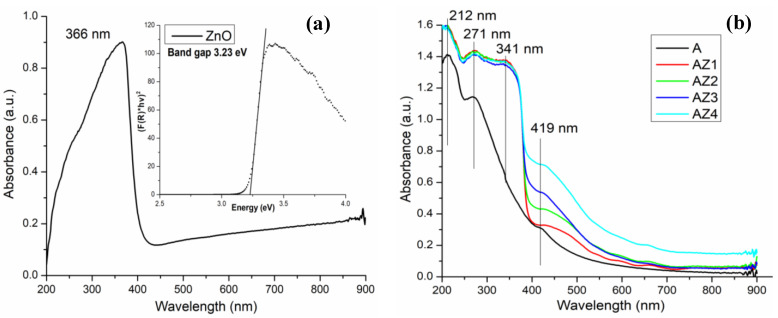
The UV-Vis spectra for ZnO nanoparticles (**a**), alginate A and AZ1–AZ4 films (**b**).

The films were light yellow from the interaction between alginate with ZnO nanoparticles and CEO. The absorption spectra of AZ1–AZ4 films are more complex than the spectra of the individual alginate and ZnO, indicating an interaction degree between the components. The absorption spectrum for the ZnO nanoparticles is presented in Figure 3a while the spectra for film samples A, AZ1–AZ4 are presented in Figure 3b. Both, the alginate and the ZnO, present UV absorption bands, with maximum at 212, 271 and 366 nm, respectively. Thus, the spectra for the AZ1–AZ4 films present a broad absorption band in the UV region, due to overlapping of individual spectra. This indicates that the films will present a robust UV shielding performance.

In the visible part of spectrum, at 419 nm, there is an absorption band, which increases in intensity as the ZnO nanoparticles content increases. This indicates the presence of alginate–ZnO interactions, as bare ZnO is white, with no visible absorption bands. This band is responsible for the increasing yellowish color of the films as ZnO content increases.

The small absorption band at 659 nm is due to the presence of citronella essential oil.

In conclusion, the films are suitable as UV barrier due to their good absorption in the UV region. The transmittance for AZ1-AZ4 films is under 5% in the 200–360 nm interval and under 10% in the 360–380 nm interval. The packaging must help preserve the organoleptic properties of the food and provide a longer shelf life. Retarding the lipid oxidation can be done by blocking the high energy wavelengths, and therefore the UV barrier property is very important in case of packaging films [79].

Due to the incorporation of ZnO NPs and CEO all the composite films have higher absorbance in the visible domain when compared with control alginate film and therefore an increased opacity [76,80]. This is similar to other reported composites with high ZnO NPs content [81]. AZ4 film has the lower transmittance (~18% at 400 nm, increasing to ~71% at 800 nm) while AZ1 film has the highest visible transmittance (~45% at 400 nm, increasing to ~88% at 800 nm). In food preservation the light barrier is also important as a way to reduce photo-oxidation of various organic compounds and degradation of vitamins or nutrients [82]. The calculated opacity values are between 0.39 and 0.85 (Table 2). These values indicate that the films are rather transparent [83], but the opacity is increasing with nanoparticles content.

#### 3.2.2. PL Spectrometry

**Figure 4:**
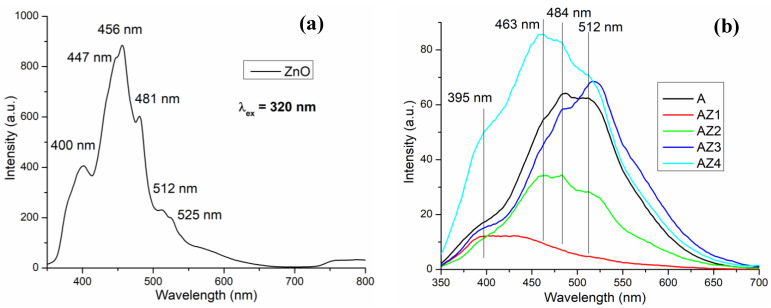
The PL spectra for ZnO nanoparticles (**a**), alginate A and AZ1–AZ4 films (**b**).

The fluorescence spectrum for the ZnO nanoparticles is presented in Figure 4a while the spectra for film samples A, AZ1–AZ4 are presented in Figure 4b. The fluorescence bands of alginate and ZnO NPs are combined into a resulting broad, but less intense single emission band, with visible shoulders in the range 390–550 nm, with an asymmetrical tail towards the higher wavelengths. Similar results are reported also in literature [84].

At low ZnO content (AZ1 and AZ2 films), the intensity of the fluorescence band of alginate is quenched, the strong ZnO emission being also blocked by the polymeric film. As ZnO quantity increases, the fluorescence emission in visible domain for AZ3 and AZ4 samples also present a limited increase. In all the samples, the fluorescence intensity remains much smaller than ZnO emission, around the alginate’s magnitude order. This indicates that the interactions between ZnO and alginate chains are blocking the recombination centers on the nanoparticle’s surface, the specific ZnO fluorescence being diminished. The ZnO fluorescence spectrum contains two distinct regions. The UV band (380–400 nm) from the free exciton recombination, and the visible region peaks which arise from various surface defects like oxygen vacancies, zinc vacancies, interstitials and antisites [18,85]. The presence of ionic polymeric matrix might offer additional centers for non-radiative recombination (at Ca^2+^ and –COO^−^), which will lead to the decrease of fluorescence intensity.

### 3.3. FTIR Spectroscopy and Microscopy

#### 3.3.1. FTIR Spectroscopy

The modification induced by the ZnO NPs and CEO to the polymeric matrix were investigated by FTIR spectroscopy. The principal absorption peaks are presented in Table 3, together with the corresponding assignment. The intense, large band from the interval 3200–3300 cm^−1^ is due to O-H bond vibrations from the alginate [86]. In the presence of ZnO the new interactions between –OH moieties and nanoparticles’ surface, induce small shifts in the peak position [87]. Such shifts were previously reported for alginate–ZnO interactions [88].

The peak from 2921 cm^−1^, attributed to C-H symmetric vibration, remains largely unchanged. Minor shifts can be observed for the peaks attributed to carboxylate symmetric and asymmetric stretching vibrations [89]. The 1027 cm^−1^ band is attributed to the glyosidic bond of the polysaccharide chain. The intense absorption peaks in the 400–500 cm^−1^ region are assigned to the presence of ZnO nanoparticles [90].

#### 3.3.2. FTIR Microscopy

**Figure 5:**
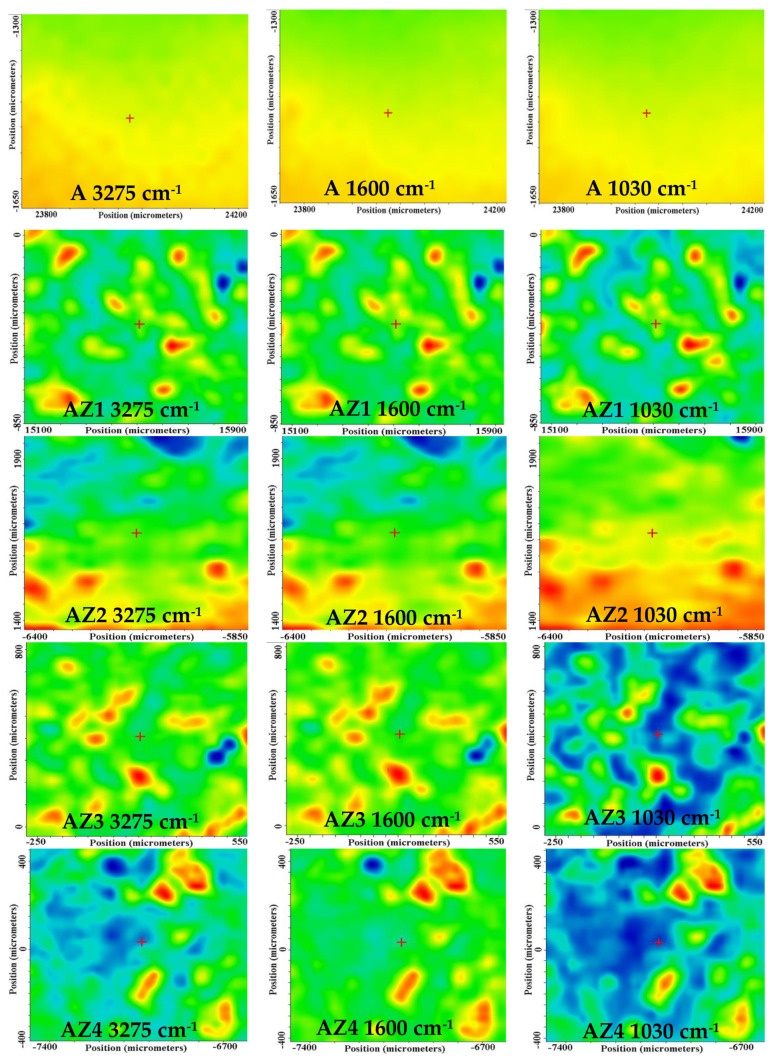
The FTIR maps for alginate (A) and AZ1–AZ4 films at wavenumbers 3275 cm^−1^ (**left column**); 1600 cm^−1^ (**middle column**); 1030 cm^−1^ (**right column**).

By means of FTIR microscopy, we were able to investigate the spatial distribution of ZnO NPs, CEO, and alginate. The FTIR maps corresponding to the strongest absorption peaks at 3275, 1600, and 1030 cm^−1^ for all five films (AZ1–AZ4 and alginate control) are presented in Figure 5. The seldom small agglomerated clusters/accumulations or defects indicate that distribution of CEO and ZnO nanoparticles on the surface of the alginate films is quite homogenous. The maps recorded at 3275 and 1600 cm^−1^ indicate that the alginate (A) and alginate/ZnO/CEO (AZ1–AZ4) films are quite homogeneous, only minor differences being present, at the level of tens of µm maximum. In the case of AZ3 and AZ4 more important heterogeneities are present as there are some noticeable differences between the maps recorded at 3275, 1600 and 1030 cm^−1^. This indicates a more heterogeneous distribution of the CEO and ZnO nanoparticles within the alginate film. ZnO can interact with CEO and a higher amount of ZnO will promote local agglomeration of nanoparticles and leave less CEO for emulsion formation.

### 3.4. Thermal Analysis

**Figure 6:**
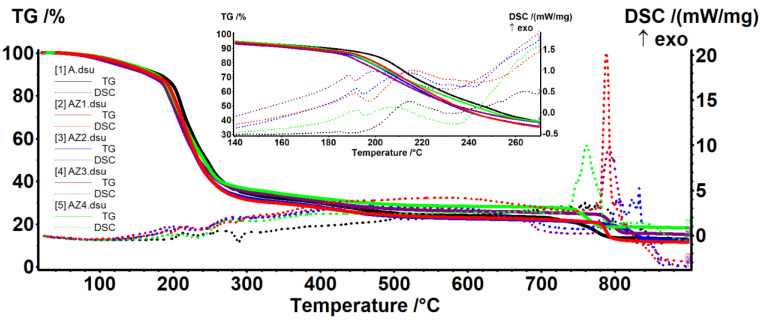
The TG-DSC curves for alginate A and AZ1–AZ4 films.

Thermal analysis curves, TG/DSC, for all composite films are presented in Figure 6 and relevant mass loss data are presented in Table 4. The presence of ZnO nanoparticles in the polymeric films does not substantially modify the thermal stability of the alginate. All AZ1–AZ4 samples start decomposing with 10–15 °C lower than the alginate control film. In the interval RT–180 °C takes place the first degradation phase, in which the samples will be dehydrated and volatile compounds from CEO will be removed [91]. This process is accompanied by a weak endothermic effect on the DSC curve.

The degradative-oxidative processes of organic part take place between 180 and 280 °C when weak exothermic effects are presented on DSC curves, low molecular weight fractions, and side chain moieties being eliminated [92]. This process may be associated with breakdown of C=O and C-C bonds from alginate chain and progressive carbonization of polysaccharide molecules [9]. Over 700 °C, the carbonaceous residue is oxidized [4], with the process being accompanied by a strong, sharp exothermic effect. At the same time, due to its inert nature vs. oxidative atmosphere, the residual mass is composed mainly from ZnO and, therefore, the highest value is for AZ4 film.

By comparing the temperatures at which the samples lost 10–50% of initial mass, a better stability can be observed for AZ4 film for most temperature intervals. The literature reports similar findings, with ZnO and TiO_2_ nanoparticles not influencing dramatically the thermal stability of the alginate films [9].

**Figure 7:**
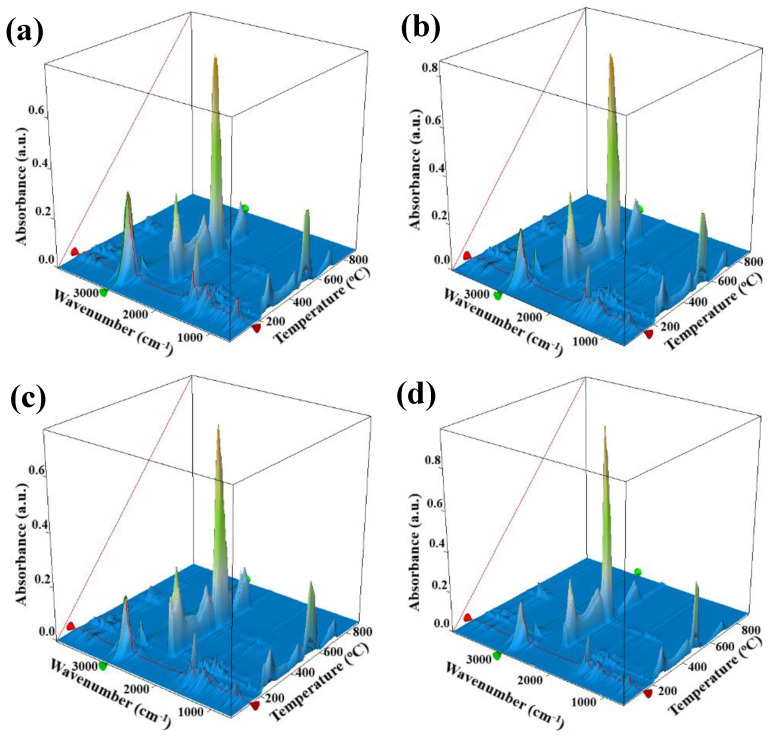
The 3D FTIR chromatogram for the evolved gases during thermal analysis for AZ1 (**a**); AZ2 (**b**); AZ3 (**c**); AZ4 (**d**) films.

The evolved gases from thermal analysis were further analyzed by FTIR to obtain a complex 3D chromatogram (Figure 7). It can be observed that at low temperatures, under 200 °C, components of CEO are eliminated from the samples (peaks at 3080, 2972 and 2926 cm^−1^ corresponding to the sp^2^ and sp^3^ C-H stretching vibrations, peak from 2725 cm^−1^ attributed to the terminal aldehydic C-H and peak at 1740 cm^−1^ attributed to C=O stretch) [93]. This indicates that some part of CEO is incorporated as it is in the alginate polymeric matrix.

Right after 200 °C components from CEO are detected again, but in smaller quantities. This indicates that by increasing the temperature the adsorbed CEO from the ZnO NPs surface begins to be eliminated. As temperature increases further any remaining CEO will be degraded together with the alginate matrix by the oxidative atmosphere.

### 3.5. Scanning Electron Microscopy (SEM)

The films’ surface morphology was investigated by analyzing the SEM images. At the same time, the energy dispersive spectrum analysis (EDS) can provide a sharp picture of the homogeneity degree of each film sample, presence of local agglomerates and nanoparticles distribution. The distribution of each element (C, O and Zn) was determined with the help of the corresponding Kα line from the X-ray spectra.

**Figure 8:**
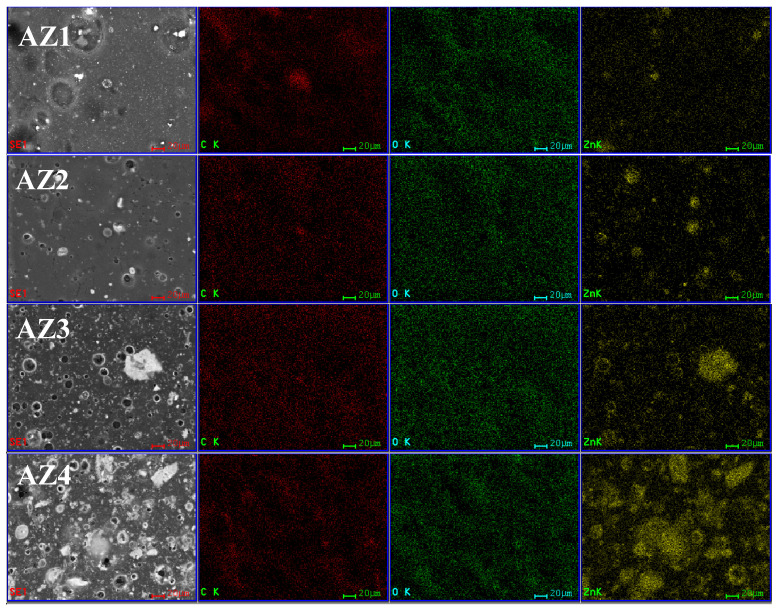
The EDS mapping images for AZ1, AZ2, AZ3, and AZ4 films (elemental distribution for C—red, O—green and Zn—yellow).

The EDS mapping images (Figure 8) indicate that the samples are rather homogenous, with some local agglomeration of nanoparticles occurring especially as the ZnO content increases. This confirms the FTIR map data about the higher inhomogeneity of AZ4 film.

**Figure 9:**
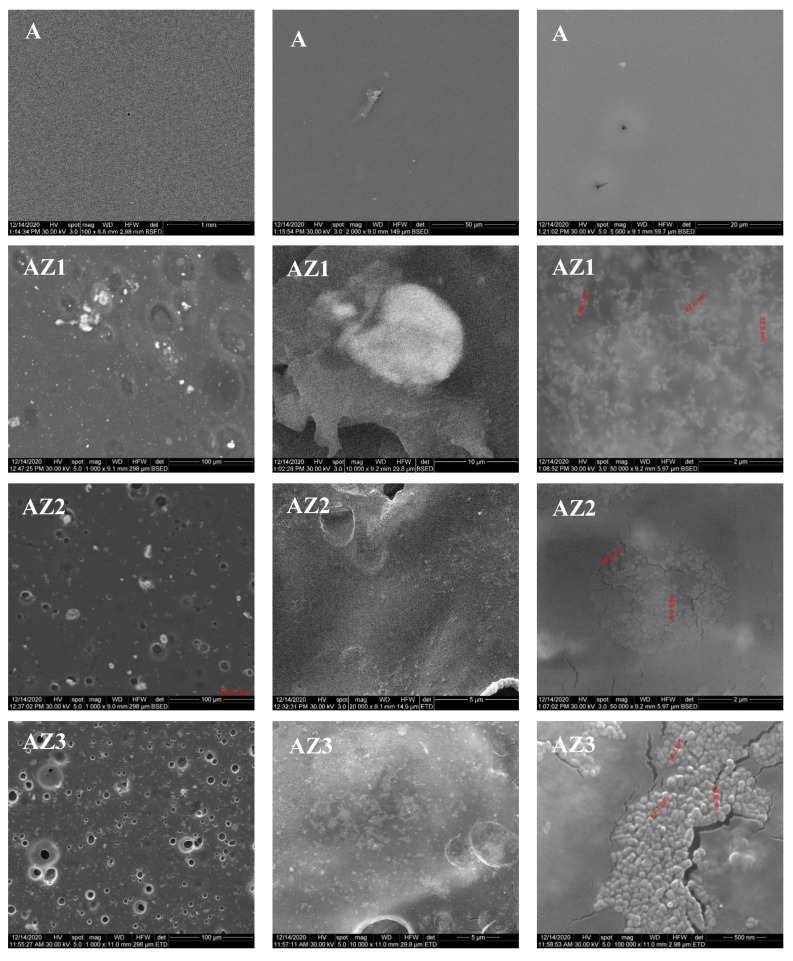

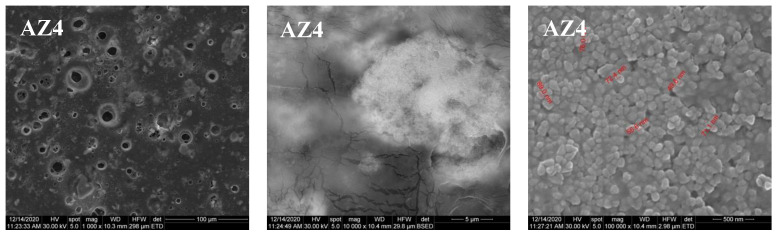
The SEM images for alginate A, AZ1–AZ4 films. On the right column, the ZnO nanoparticles were measured (red numbers).

The SEM images for the alginate control film, Figure 9, present a uniform surface. Higher magnifications indicate the presence of some minor defects, like pores. For the AZ1-AZ4 films containing alginate/ZnO/CEO, the SEM images are also presented in Figure 9. The SEM images indicate the presence of some pores on the surface of all films. The size of the pores decreases as the ZnO amount increases, most probably because the ZnO nanoparticles adsorb the CEO better and there are smaller quantities of oil to form the emulsion in alginate solution. When ZnO amount is small, there are many droplets of CEO in the alginate solution, which can coalescence to form larger pores on the film surface. Consequently, the pore size decreases when ZnO amount increases. The large size pores are presented on the AZ1 film, while for the AZ2 sample the pore size decreases sharply to smaller diameters, even less than 5 μm, but with a homogeneous distribution. The water vapor permeability measurements indicate a better performance for the films with higher ZnO content. As the pores observed on the surface, in the SEM images, are not connected and do not cross the membrane it seems that their influence is negligible. At the same time, the increasing amount of ZnO leads to denser membranes, with lower permeability. The higher magnification SEM images (50–100 k X) reveal the presence of ZnO nanoparticle agglomerates in the alginate film structure, with sizes in the 48–89 nm interval (right column images from Figure 9).

### 3.6. Water Vapor Permeability (WVP)

Ideally, loss of water, flavor and other specific substances should be negligible through packaging films for food [92]. Water vapor permeability (WVP) values are important as it controls the moisture migration between food and the outside of packaging. Usually microbial spoilage of food can be associated also with a high value for WVP.

The obtained values for WVP determination are presented in Table 5. The WVP value for the alginate control (A) film is average when compared with literature [92,94] due to addition of glycerol, as this will form hydrogen bonds with the base polymer, which further increase the chain–chain distance [95]. Nevertheless, important decreases in WVP are observed for all samples when compared with alginate control film. The high decrease between A and AZ1 can be explained by the presence of both CEO and ZnO. CEO has a clear hydrophobic nature and therefore will act towards decreasing of WVP, similar findings being reported [13].

**Figure 10:**
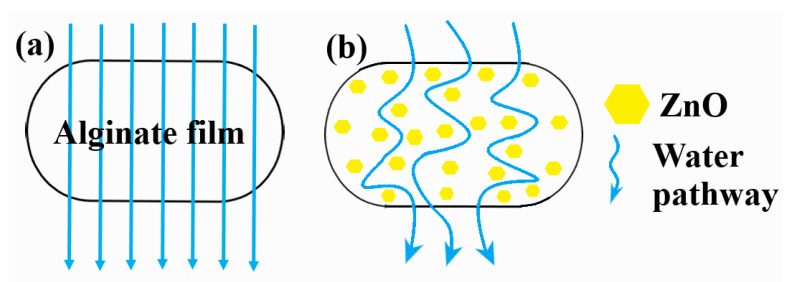
The proposed mechanism behind decrease of WVP values for AZ1–AZ4 films. Water pathway through the Alginate film (**a**) and through AZ1-AZ4 films (**b**).

The presence of ZnO nanoparticles also act as a physical barrier, increasing the pathways for water molecules [5,92]. The values for WVP decreased as the content of ZnO NPs increased. This decrease of WVP values can be due to the formation of ZnO–alginate composite structure, in which tighter and denser arrangements appear, with ZnO nanoparticles being impermeable to water. As such, the water has a more complicated, longer pathway through these composite films, as presented in Figure 10.

Further decrease of WVP values for AZ2–AZ4 films, as ZnO amount increases, confirms the influence of mineral nanoparticles. The increase in SD values can also be ascribed to the increasing presence of local agglomeration of nanoparticles, which equals with decreasing homogeneity, as seen in SEM images.

A higher value for WVP can be expected from a porous structure, when the pores are connected. As the SEM micrographs indicate, the AZ1–AZ4 films have a porous surface, but the pores are not connected and do not cross the membrane.

### 3.7. Swelling Study

The swelling studies were carried out in both water and PBS (pH = 7.4). The obtained data, Table 6, indicate a decrease of the swelling capacity with the increase of ZnO content in the alginate film.

**Figure 11:**
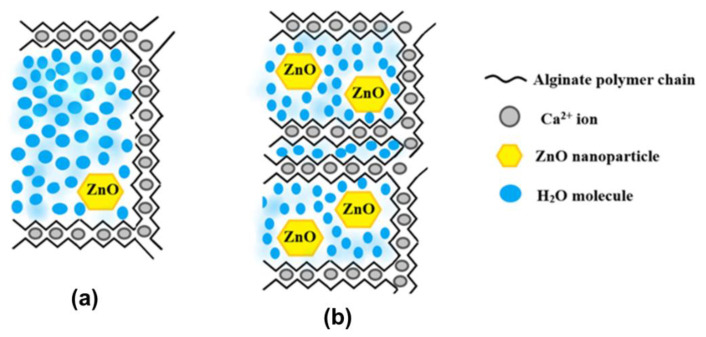
The swelling process for alginate-ZnO and CEO films (AZ1–AZ4). Films with low ZnO nanoparticles content can uptake more water (**a**). Higher ZnO nanoparticles content will inhibit water uptake, resulting in a lower swelling percentage (**b**).

The AZ1–AZ4 films presented a lower water uptake capacity, as the ZnO amount in each composition increases. The proposed mechanism is presented in Figure 11. As more ZnO nanoparticles are present in the AZ films, the alginate chains will interact with them, and will lock them in closer positions, with smaller gaps available for water molecules. Additionally, as there is an important difference between the water absorption capacity of alginate and ZnO, thus a higher amount of ZnO will decrease the hydration capacity. Therefore, the swelling capacity will decrease with the increase of ZnO nanoparticles number. These results are in agreement with the WVP data.

All the films presented a fast water uptake, as the close to maximum swelling capacity was obtained after 30 min. The swelling continued to increase slowly for 3 h, but at 24 and 48 h the mass of the films begins to decrease, the mass loss indicating a possible solubilization of some alginate from the samples.

The values obtained in the PBS swelling study were larger if compared with those obtained in the water swelling study. In addition, the alginate films in PBS start to present noticeable disintegration after 48 h, while those kept in water are keeping their integrity even after 4 weeks. This can be explained by the slow replacing of Ca^2+^ ions with Na^+^. As the process advances, the “egg-box” model of calcium-alginate interactions is replaced by the simple sodium-alginate formation, which is water soluble. The chains begin to break apart and the films disintegrate.

### 3.8. Antibacterial Activity

The evaluation of the growth inhibition results determined that the AZ1-AZ4 films show a different antibacterial effect, depending on the tested strain and ZnO NPs content. We utilized Gram-positive (*S. aureus*; *B. cereus*) and Gram-negative (*S.* Typhi; *E. coli*) strains, relevant in foodborne infections, in order to highlight antibacterial coverage of the obtained nanomodified films. The control alginate film presented no antibacterial activity.

**Figure 12:**
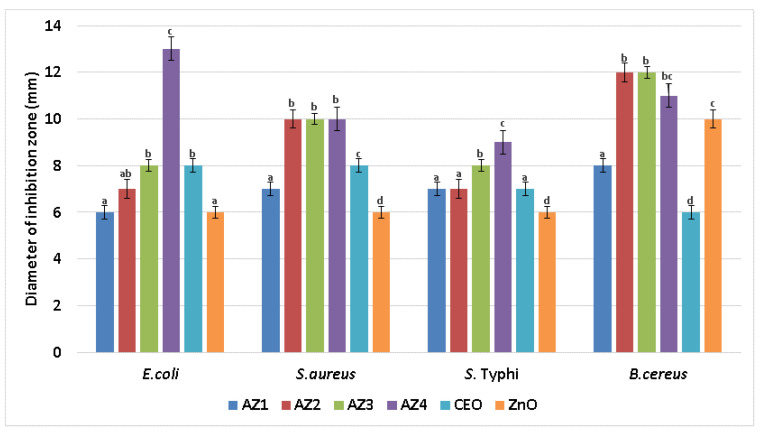
Graphic representation of growth inhibition diameters (shown in mm) obtained after the cultivation of evaluated bacterial strains in the presence of alginate and AZ1–AZ4 films. Different small letters indicate statistically significant differences between films (*p* < 0.05).

Results show that growth inhibition is impaired in a ZnO concentration manner in all of the investigated strains, the highest values of growth inhibition zones being obtained for AZ4 samples, followed by AZ3, then AZ2 and AZ1. Figure 12 also reveals that the presence of CEO has a synergic antibacterial effect, together with ZnO. Plain CEO shows a lower growth inhibition potential, but this inhibition is increased in ZnO containing samples. Intriguingly, the ZnO control samples showed a modest antibacterial effect, this being significant only in *B. cereus* strain. Therefore, these results suggest that synergistic activity of CEO and ZnO is responsible for growth inhibition in evaluated strains (Figure 12).

**Figure 13:**
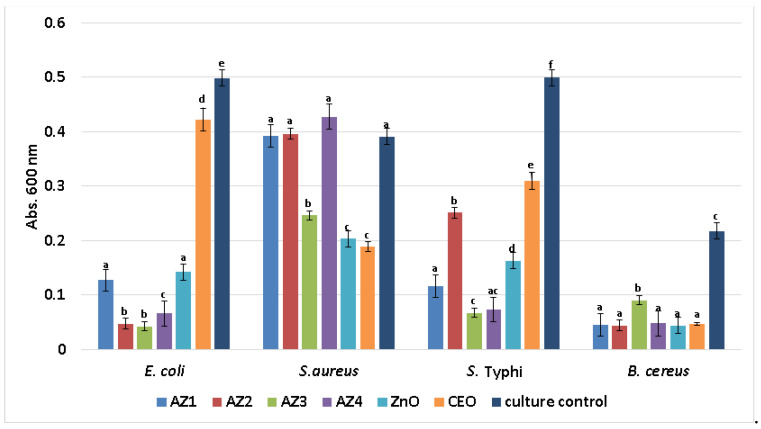
Graphic representation of average absorbances at 600 nm revealing growth of planktonic bacterial cultures in the presence of control and AZ1-AZ4 films for 24 h at 37 °C. Different small letters indicate statistically significant differences between films (*p* < 0.05).

Planktonic growth inhibition results showed that bioactive compounds (i.e., CEO and ZnO NPs) could be released from the nanostructured materials, since they affect the development of free-floating cells. Figure 13 reveals that the highest bacterial growth inhibition in nutritive broth is obtained for the samples containing CEO and higher amounts of ZnO NPs. However, growth inhibition was also obtained for ZnO NPs and CEO controls, but the results are less significant.

Although the initial amount of CEO in the AZ1–AZ4 films is equal, its distribution is not identical. Part of the CEO is loaded on the ZnO NPs surface and the rest is dispersed inside the film thickness as droplets. As the ZnO NPs content increases, the amount of CEO adsorbed on the nanoparticles surface increases, and the free CEO inside the film decreases. Therefore, while in AZ1 film a larger CEO amount is available from the beginning, in AZ4 film a longer release time for CEO is expected.

Not only the growth of planktonic cells was influenced by the obtained AZ1-AZ4 films, but also their attachment and biofilm development. Our data show biofilms are significantly reduced after 24 h incubation in samples containing CEO and ZnO NPs, in a ZnO NPs concentration dependent manner.

**Figure 14:**
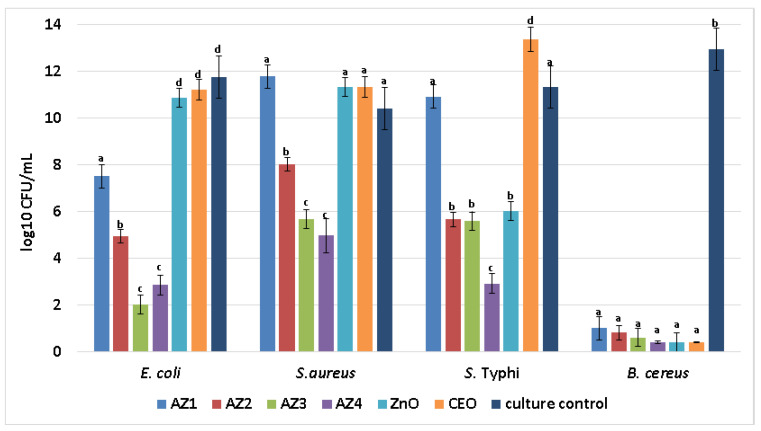
Graphical representation of log10 CFU/mL values obtained for the tested Gram-positive and Gram-negative bacterial strains, expressing biofilm embedded cells developed on control and AZ1-AZ4 films after 24 h incubation. Different small letters indicate statistically significant differences between films (*p* < 0.05).

Highest biofilm inhibition was obtained in the presence of AZ4, followed by AZ3 samples, the inhibition being relatively uniform among all tested bacterial isolates (Figure 14). However, the highest antibacterial effect was seen against the Gram-positive *B. cereus* microorganism, suggesting these coatings may be tailored to target particular types of bacteria, depending on the intended application and on the susceptibility of each strain.

The literature indicates that CEO is especially effective against Gram-positive bacterial strains like *S. aureus* or *B. cereus* [96,97]. At the same time, ZnO nanoparticles are more potent against *E. coli* than against *S. aureus* [18,19]. Nonetheless, our studies reveal that these alginate/ZnO/CEO films have a good antibacterial coverage, the ZnO nanoparticles and CEO acting synergic, thus being efficient against several pathogens, despite Gram-negative strains being usually more resistant than Gram-positive due to their intrinsic resistance mechanism [96,98].

### 3.9. Preliminary Evaluation of AZ1–AZ4 Films as Coatings for Soft Cheese

**Figure 15:**
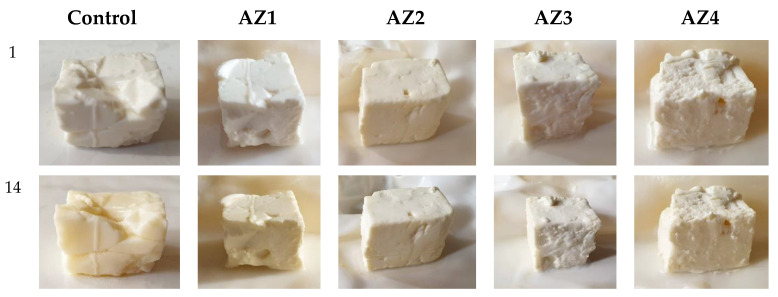
Visual appearance of soft cheese bits packaged in alginate control film and AZ1–AZ4 films, initial and after 14 days storage at 4 °C and 75% relative humidity.

Soft cheese was packed in the alginate composite films (containing ZnO NPs loaded with CEO) and was stored at 4 °C ± 1 °C and 75% relative humidity (RH) for 14 days. A control lot was packed in the simple alginate film. The preliminary visual quality check (Figure 15) and weight loss data (Table 7) indicates that AZ1–AZ4 films were effective in preserving the cheese.

The samples packed in AZ1–AZ4 films were clearly different from the control sample after 14 days. While the control sample was rather hard and translucent with visible signs of spoilage, the AZ1–AZ4 packaged samples were whiter, soft and kept the original creamy texture of the cheese.

The weight loss of the cheese bits increased linearly, in all cases, with storage time, with average values ranged from 2.15% for control sample to 0.43% for AZ4 packed sample (Table 7). This is possible because of the continuous water vapor movement from cheese to surrounding environment.

The pH value of the cheese was monitored daily and was found to be in the interval 4.45–4.57 for all AZ1–AZ4 samples, indicating little to no variation. The quasi constant pH value of the coated cheese is reported previously in literature [99]. However, the pH of the control sample dropped to 4.33, probably due to increased fermentation promoted by microorganisms.

## 4. Conclusions

Innovative biodegradable packaging films based on alginate were obtained. The antibacterial activity was ensured by the addition of ZnO nanoparticles loaded with CEO. The films are transparent, but the opacity increases with the ZnO content. Even at the highest ZnO content, the films can be considered homogenous, with no clefts. The presence of ZnO and CEO in the alginate films has improved the light and water barrier properties. The antibacterial assay indicates that the inhibition is relatively uniform among all four bacterial tested strains. The best antibacterial activity is reported for *B. cereus*. Overall the ZnO and CEO acts in a synergic way, strongly inhibiting the growth of both Gram-positive and Gram-negative strains. Preliminary test on soft cheese indicates that the samples preserved in AZ1–AZ4 films retained the color, surface texture and softness over 14 days, successfully extending the shelf life, while weight loss decreased as ZnO content increased. In conclusion, an antibacterial potential packaging for cheese and other foods based on alginate/ZnO/CEO was for the first time obtained and characterized.

## Figures and Tables

**Table 1:** The alginate–ZnO NPs–citronella essential oil (CEO) films composition.

Sample Code	Alginate (g in 100 mL Water)	ZnO NPs (g in 15 mL Water)	Glycerol (mL Solution)	CEO (mL)
A	3.00	0.00	2	0
AZ1	3.00	0.05	2	1
AZ2	3.00	0.10	2	1
AZ3	3.00	0.25	2	1
AZ4	3.00	0.50	2	1

**Table 2:** Transmittance at 200–800 nm, thickness (mm) and opacity for alginate (A) and alginate/ZnO/CEO (AZ1–AZ4) films.

Sample	Transmittance (%)							Thickness (mm)	Opacity
	200 nm	300 nm	400 nm	500 nm	600 nm	700 nm	800 nm		
A	4.42	10.87	45.76	72.74	85.27	91.07	94.09	0.25 ± 0.02	0.28 ± 0.02 ^a^
AZ1	2.50	4.06	44.60	59.16	79.79	88.27	87.67	0.24 ± 0.06	0.39 ± 0.09 ^a,b^
AZ2	2.44	4.01	34.98	50.76	73.84	85.11	85.95	0.30 ± 0.07	0.45 ± 0.09 ^b^
AZ3	2.60	4.27	26.52	50.34	75.75	86.45	88.42	0.24 ± 0.03	0.50 ± 0.06 ^b^
AZ4	2.35	4.16	18.08	33.72	56.58	68.21	70.86	0.29 ± 0.03	0.85 ± 0.07 ^c^

Different superscripts (a, b, c) in the last column are significantly different (*p* < 0.05). Values are given as mean ± SD from triplicate determination.

**Table 3:** Assignment of relevant IR absorption bands of alginate (A) and AZ1–AZ4 films.

Sample/Assignment	A	AZ1	AZ2	AZ3	AZ4
υZn-O		426	427	428	429
		473	474	467	470
υ_as_C-O-C	1027	1027	1028	1028	1027
υ_s_COO^−^	1408	1408	1409	1409	1408
υ_as_COO^−^	1600	1602	1602	1602	1601
C=O group of CEO [41]		1740	1737	1738	1737
υC-H (sat)	2932	2924	2920	2922	2922
υO-H	3263	3273	3273	3273	3275

**Table 4:** Temperatures at which alginate and AZ1–AZ4 films lost 10%, 20%, 30%, 40%, or 50% of initial mass.

Sample	T_10_	T_20_	T_30_	T_40_	T_50_	Residual Mass
A	180	207	216	229	245	12.77%
AZ1	171	200	211	222	235	11.37%
AZ2	165	198	209	220	233	12.28%
AZ3	165	194	206	219	235	15.01%
AZ4	176	198	211	225	242	18.15%

**Table 5:** Water vapor permeability (WVP) for control alginate (A) and AZ1–AZ4 films.

Film Code	WVP (10-10 g/Pa∙m∙s)
A (alginate control)	5.718 ± 0.011 ^a^
AZ1	4.542 ± 0.018 ^b^
AZ2	4.126 ± 0.023 ^c^
AZ3	3.687 ± 0.056 ^d^
AZ4	3.043 ± 0.086 ^e^

Different superscript letters indicate statistically significant differences between films (*p* < 0.05).

**Table 6:** Swelling percentage for the alginate and AZ1–AZ4 films over 48 h.

Sample	Water PBS					
	0.5 h	1 h	2 h	3 h	24 h	48 h
A	552.29%	578.36%	596.51%	616.29%	600.99%	590.38%
	539.12%	652.05%	701.89%	719.90%	742.41%	739.26%
AZ1	516.83%	536.86%	537.98%	550.79%	538.94%	534.39%
	604.69%	704.03%	745.48%	756.20%	789.36%	672.18%
AZ2	484.78%	477.38%	528.66%	542.09%	522.53%	528.36%
	456.87%	634.35%	708.18%	723.69%	762.62%	750.84%
AZ3	427.03%	450.41%	417.68%	434.96%	421.95%	427.24%
	360.64%	530.95%	640.04%	655.10%	703.20%	688.63%
AZ4	297.27%	342.73%	379.77%	398.08%	394.69%	354.96%
	282.05%	374.36%	595.63%	659.27%	741.75%	684.43%

**Table 7:** Weight loss for cheese bits coated with alginate control and AZ1–AZ4 films during storage.

Sample	Weight Loss (%)				
	1 Day	4 Days	7 Days	10 Days	14 Days
A	2.01 ± 0.15 ^a^	8.16 ± 0.12 ^a^	14.56 ± 0.16 ^a^	21.21 ± 0.18 ^a^	30.10 ± 0.23 ^a^
AZ1	1.81 ± 0.09 ^a^	7.32 ± 0.11 ^b^	13.02 ± 0.12 ^b^	18.91 ± 0.15 ^b^	26.74 ± 0.19 ^b^
AZ2	1.27 ± 0.05 ^b^	5.16 ± 0.04 ^c^	9.31 ± 0.07 ^c^	13.70 ± 0.11 ^c^	19.46 ± 0.12 ^c^
AZ3	0.86 ± 0.06 ^c^	3.56 ± 0.06 ^d^	6.51 ± 0.10 ^d^	9.50 ± 0.11 ^d^	13.72 ± 0.15 ^d^
AZ4	0.33 ± 0.03 ^d^	1.41 ± 0.04 ^e^	2.66 ± 0.08 ^e^	4.11 ± 0.07 ^e^	6.02 ± 0.11 ^e^

Different superscript letters in the same column indicate statistically significant differences between films (*p* < 0.05).

## Data Availability

Not applicable.
